# Size does not matter: molecular phylogeny reveals one of the largest trematodes from vertebrates, the enigmatic *Ithyoclinostomum dimorphum*, as a species of *Clinostomum* (Trematoda: Clinostomidae)

**DOI:** 10.1016/j.ijppaw.2022.08.002

**Published:** 2022-08-24

**Authors:** Mariana B. Simões, Philippe V. Alves, Danimar López-Hernández, Elimayke A. Couto, Narcisa I.B. Moreira, Hudson A. Pinto

**Affiliations:** a Laboratório de Biologia de Trematoda, Instituto de Ciências Biológicas, Universidade Federal de Minas Gerais, Belo Horizonte, MG, Brazil; b Laboratório de Helmintologia Animal, Departamento de Patologia, Universidade Federal do Espírito Santo, Vitória, ES, Brazil

**Keywords:** Trematodes, Phylogeny, Taxonomy, Clinostomid, Birds, Helminths

## Abstract

Despite the recent advances raised in the molecular era to the taxonomic knowledge of species of the family Clinostomidae, especially those belonging to the specious genus *Clinostomum*, some groups of these vertebrate parasites remain poorly studied. This is the case of species of the enigmatic genus *Ithyoclinostomum* Witenberg, 1926, until recently monotypic and restricted to South America, but with its occurrence expanded to North America after the description of *I*. *yamagutii* Rosser et al., 2020. Nevertheless, molecular data for the type species of the genus, *Ithyoclinostomum dimorphum* (Diesing, 1850), is lacking so far. In the present study, large clinostomid metacercariae morphologically indistinguishable from *I. dimorphum* were obtained from two erythrinid fishes from the Rio Doce River, Southeast Brazil. Samples of the parasites were subjected to a multigene (28S rDNA, ITS and *cox*1) molecular characterization followed by phylogenetic reconstructions. Phylogenies based on single-gene and concatenated datasets revealed unequivocally that *I. dimorphum* falls in a well-supported clade together with species of the genus *Clinostomum*. Moreover, the molecular divergences observed in relation to *Clinostomum* spp. [ranges of 2.4–6%, 2.4–3.8% and 14.7–19.3% for the ITS, 28S and *cox*1 genes, respectively] are compatible with a congeneric status with these species. Therefore, the genus *Ithyoclinostomum* is here synonymized with *Clinostomum* Leidy, 1856 and *C. dimorphum* (Diesing, 1850) Braun, 1899 re-established. In the phylogenetic analysis, the recently described '*Ithyoclinostomum*' *yamagutii*, presented as an isolated, independent lineage, showing significant molecular divergences to *C. dimorphum* (12.6%, 7.6%, 18,6% for the ITS, 28S and *cox*1 genes, respectively). However, given the complex scenario raised in the morphology-based taxonomy of Clinostomidae, we took a conservative approach by not proposing a new genus to '*I*.' *yamagutii* until molecular data of other clinostomid genus from birds, *Clinostomatopsis*, become available. Data here presented reveals that body size is not a useful criterion for higher-level classification in Clinostomidae. Finally, we highlighted the importance of the availability of molecular data for the type species of trematode genera proposed from South America to support a trans- or intercontinental distribution.

## Highlights

• One of the largest trematodes ever known was sequenced for the first time.
• Despite remarkable difference in body size, sequenced specimens belong to *Clinostomum*.
• *Ithyoclinostomum* is synonymized with *Clinostomum* and *C. dimorphum* re-established.
• Data presented pave the way towards a more natural classification of Clinostomidae.
• Sequences for type-species of trematode genera described from South America is needed.

## Graphical abstract

**Figure:**
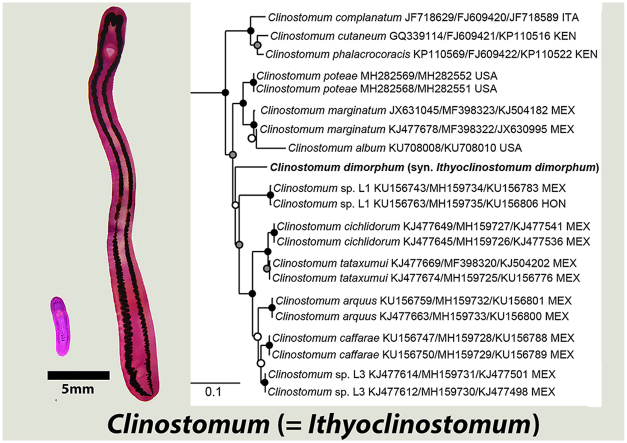

## 1 Introduction

Members of the family Clinostomidae Lühe, 1901 are digenetic trematodes found, in the adult stage, in the oral cavity and oesophagus, mainly from birds and reptiles but occasionally in mammals, including humans (Kanev et al., 2002). More than 30 species have been described worldwide, resulting in a complex taxonomy revised by different authors over time (Braun, 1901; Ukoli, 1966; Kanev et al., 2002). The use of molecular data has allowed important advances in the knowledge of this group, including the description of new species, the link between larval stages and adults and inferences about the phylogeny, ecology and biogeography of clinostomids (e.g., Caffara et al., 2011, 2019; Locke et al., 2015, 2019; Woodyard et al., 2017; Rosser et al., 2017, 2020; Sereno-Uribe et al., 2018). However, a proper classification that reflects the evolutionary history of the group is still in progress and far from being achieved, mainly because molecular data for some key taxa (type genera and species) is lacking. In fact, most information is available only for the species-rich *Clinostomum* (Supplementary Table S1), including numerous unnamed lineages of this genus that are awaiting their formal description (Montes et al., 2021).

Among the poorly studied clinostomids is the until recently monotypic genus *Ithyoclinostomum* Witenberg, 1926, which includes species that adult forms parasitize the oesophagus of fish-eating birds. The type species of this genus was originally described as *Distoma dimorphum* by Diesing (1850) from ardeids in Brazil. Since then, a complex taxonomic history began to unfold. First, different species were found in the material studied by Diesing (1850), one of which was included in the genus *Clinostomum* (as *Clinostomum dimorphum*) by Braun (1901). Then, years later, *C. dimorphum* was allocated in a new genus, *Ithyoclinostomum* Witenberg, 1926, typified under its impressive size, reaching up to 10 cm. Since then, the finding of birds and fish harboring very large clinostomids identified as *I. dimorphum* became common in South America, and most reports were from Brazil (for a detailed list of host, localities and reports, see Briosio-Aguilar et al., 2019). To the best of our knowledge, these are the largest trematodes found in these host groups of vertebrates [considering the respective developmental stage – adult from birds and metacercariae from fish (mainly erythrinids)]. This peculiar morphological trait has been considered diagnostic for the genus and used, for instance, to typify the subfamily Ithyoclinostominae by Yamaguti (1958). This size-based classification was maintained in the most recent taxonomic review of the group (Kanev et al., 2002).

Despite advances in the phylogenetic classification of trematodes, especially Clinostomidae, there are still no sequences available to *I. dimorphum*, the type species of the genus *Ithyoclinostomum*. More recently, the second species of the genus, *Ithyoclinostomum yamagutii* Rosser et al., 2020, was described from birds and linked to metacercariae from fish in North and Middle America (Briosio-Aguilar et al., 2019; Rosser et al., 2020). Again, the large size (∼25 mm; yet much smaller than *I*. *dimorphum*) was one of the differential traits for including the specimens in the genus *Ithyoclinostomum*. Although robust phylogenetic data have revealed *I. yamagutii* as distinct from other clinostomid genera, the congeneric status with *I. dimorphum* still needs to be confirmed. Moreover, the systematic value of this impressive large-sized body, traditionally used in the clinostomid classification, remains an open question for investigation. In the present study, the phylogenetic position of *I. dimorphum* was evaluated for the first time and the results revealed it as a member of *Clinostomum*.

## 2 Material and methods

During a long-term project aimed at evaluating the helminth fauna of fish from the Rio Doce, in its portion in the state of Espírito Santo, Southeast Brazil, large clinostomid metacercariae were collected from *Hoplias malabaricus* (Bloch) and *Hoplias intermedius* (Günther), between December 2020 and July 2021. Metacercariae were found encysted and so mechanically excysted, ringed in physiological saline, gently compressed dorsoventrally between two glass slides, killed with hot water and fixed in 10% formalin. Subsequently, the helminths were stained with alum acetocarmine, dehydrated in ethanol series, diaphanized in beechwood creosote and mounted in Canada balsam.

**Fig. 1:**
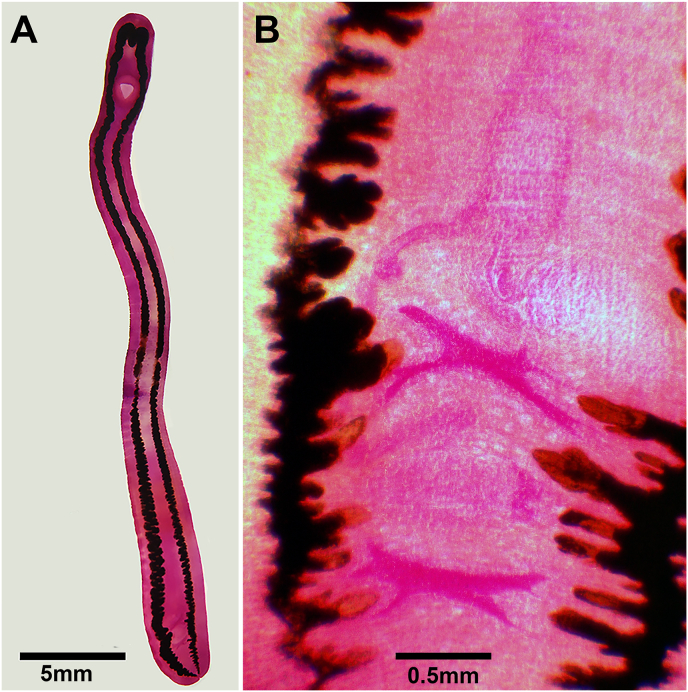
Metacercariae of *Clinostomum dimorphum* found in the erythrinid fish, *Hoplias intermedius* from Brazil: (A) Whole view of a paragenophore specimen. B) Detail of reproductive structures of a hologenophore specimen.

**Supplementary Fig. S1:**
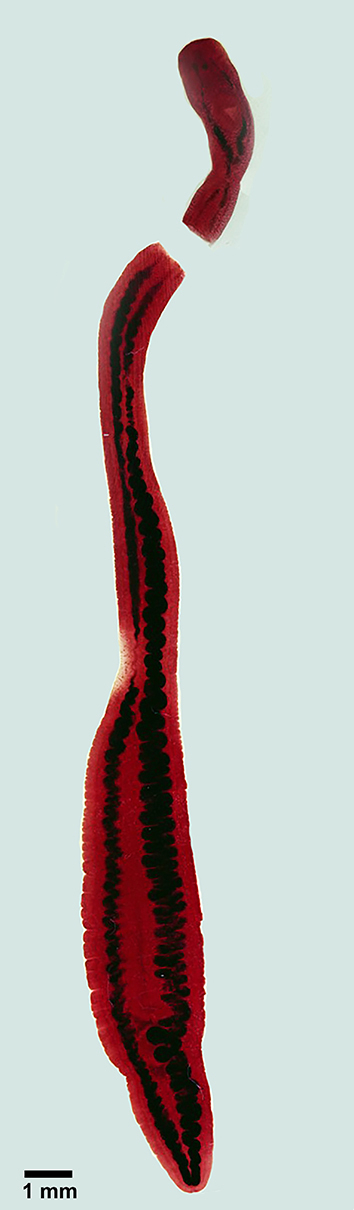
Whole view of a hologenophore specimen of *Clinostomum dimorphum* (= *Ithyoclinostomum dimorphum*) found in *Hoplias malabaricus* from Brazil.

Two hologenophores, i.e., vouchers from the same specimens from which molecular data was gathered (one from each host species), were sequenced for the molecular study (Fig. 1, Supplementary Fig. S1). DNA extraction of ethanol fixed worms was made using the QIAamp® DNA Mini kit (Qiagen, USA), according to manufacturers' instructions. The concentration and quality of extracted DNA were evaluated using the microvolume spectrophotometer NanoDrop® Lite (Thermo Fisher Scientific, USA). Partial regions of genes 28S (primers Dig-12/1500R), ITS1-5.8S-ITS2 (primers BD1/BD2), and *cox*1 (primers Dice-1 and Dice-11) were amplified by PCR, using the conditions previously described (Tkach et al., 2003; Morgan and Blair, 1995; Van Steenkiste et al., 2015). PCR reagents, electrophoresis, amplicons purifications and sequencing were as previously described in the works of our research group (Alves et al., 2020). Chromatograms obtained were assembled and inspected for errors in ChromasPro v.2.0.1 software (Technelysium Pty Ltd, Australia) and consensus sequences used for phylogenetic analyses together with other members of Clinostomidae available in GenBank.

The sequences generated *de novo* were assembled into four alignments using default parameters of MAFFT algorithm (Katoh and Standley, 2013) implemented in GUIDANCE server (Sela et al., 2015) as follows: (i) including representatives of all species/species-level lineages with available sequences, assembled in three alignments according to each molecular marker (ITS, 28S and *cox*1), (ii) using only representatives with available sequences for all markers (concatenated dataset). Unreliable positions in the single-gene alignments were identified and removed using the Gblock web server (https://ngphylogeny.fr/; Dereeper et al., 2008) with less stringent settings, except for the *cox*1 dataset where no indels (insertions/deletions) were found; no gaps were allowed in the Gblock settings for the concatenated dataset. Missing ends in the alignments were replaced with 'N's.

Phylogenetic reconstructions were performed with the maximum likelihood (ML) criterion using the evolutionary models implemented in ModelFinder (Kalyaanamoorthy et al., 2017) within IQTREE (Trifinopoulos et al., 2016), based on the small sample size corrected Akaike information criterion (AICc). The models used were as follows: TVM+F+G4 for both ITS-1 and ITS2, and K2P+I for 5.8S, all included in the ribosomal gene cluster ITS1-5.8S-ITS2 alignment (3 partitions); TVM+F+I+G4 for the 28S dataset alone; TIM3+F+I+G4, TIM+F+G4, GTR+F+G4, for the 1st, 2nd and 3rd codon positions of the *cox*1 dataset, respectively (3 partitions); GTR+F+G4, K2P+I, TVM+F+G4, TVM+F+I+G4, TN+F+G4, K3Pu+F+G4, TPM2+F+G4, for the ITS1, 5.8S, ITS2, 28S, *cox*1 1st + 2nd + 3rd codon positions of the concatenated dataset (7 partitions). Outgroups were chosen based on previous phylogenetic studies (Briosio-Aguilar et al., 2019; Rosser et al., 2020; Montes et al., 2021).

The ML trees were generated via IQTREE and clade supports were estimated with 10,000 replicates of the ultrafast bootstrap (UFBoot—Minh et al., 2013) and an SH-aLRT test with 10,000 replicates (Guindon et al., 2010). Clades with support values of both UFBoot ≥95 and SH-aLRT ≥ 80 were considered strongly supported, while clades with only one of UFBoot ≥95 or SH-aLRT ≥ 80 were weakly supported; nodes with both UFBoot <95 or SH-aLRT < 80 were unsupported. All the above-mentioned analyses were run on the computational resource CIPRES (Miller et al., 2010). Uncorrected *p*-distances were calculated using MEGA 7.0 (Kumar et al., 2016). Newly generated sequences were deposited in the GenBank [OP171941 (28S); OP171939, OP171940 (ITS); OP174427, OP174428 (*cox*1)].

## 3 Results and discussion

The morphological and morphometric analyses were performed under a light microscope, and the data obtained were compared with information reported or compiled by different authors (Kanev et al., 2002; Briosio-Aguilar et al., 2019). The morphology and measurements of metacercariae studied were indistinguishable from *I. dimorphum* (Fig. 1, Supplementary Table S2). As in other clinostomids, metacercariae were found well-developed, including morphological traits of the reproductive system, enabling the link with the adult stage. Since *I*. *dimorphum* is morphologically well-characterized, detailed morphological analysis is not included in the present account, instead, readers are referred to Briosio-Aguillar et al. (2019), and references therein, for a more exhaustive morphological treatment.

The ribosomal cluster alignment (ITS1-5.8S-ITS2) included 33 sequences (two as outgroup) comprising 981 positions in the final dataset, while the other nuclear marker, 28S, included 30 sequences (one as outgroup) totaling 1245 positions. The mitochondrial *cox*1 alignment included 56 sequences (two as outgroup) comprising 798 positions in the final dataset. The concatenated alignment of the partitioned ITS + 28S + partitioned *cox*1 included 29 sequences (one as outgroup) comprising 2532 positions.

**Fig. 2:**
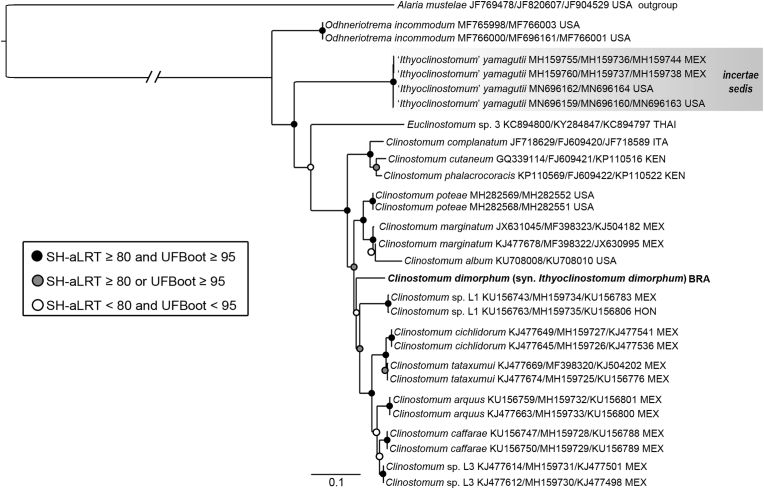
Maximum likelihood phylogram based on the concatenated ITS1-5.8S-ITS2 + 28S + *cox*1 datasets of *Clinostomum dimorphum* (in bold) and selected species of the family Clinostomidae. Clade formed by isolates of ‘*Ithyoclinostomum*’ *yamagutii* (*incertae sedis*) is highlighted in grey. Taxon names are followed by GenBank accession numbers of ITS, 28S, and *cox*1, respectively, and country of record. Branch length scale bar indicates number of substitutions per site. Abbreviations: HON, Honduras; ITA, Italy; KEN, Kenya; MEX, Mexico; THAI, Thailand; USA, United States of America.

**Supplementary Fig. S2:**
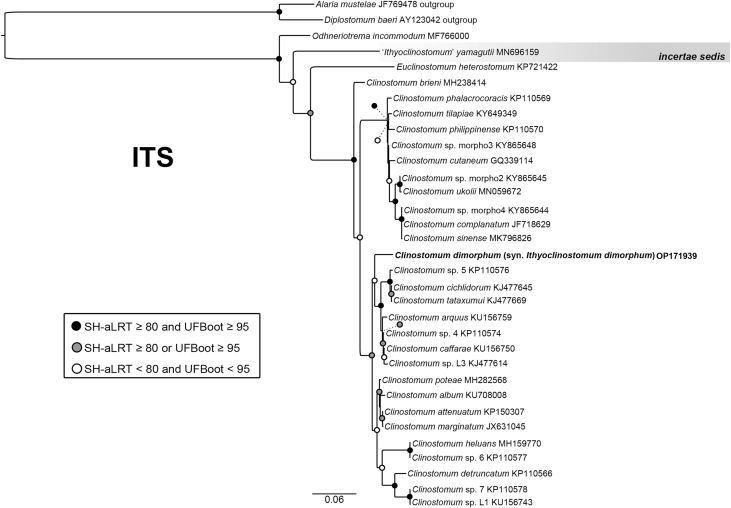
Maximum likelihood phylogram based on the ITS1-5.8S-ITS2 dataset of *Clinostomum dimorphum* (in bold) and selected Clinostomidae. Isolate of ‘*Ithyoclinostomum*’ *yamagutii* (*incertae sedis*) is highlighted in grey. Taxon names are followed by GenBank accession numbers. Branch length scale bar indicates number of substitutions per site.

**Supplementary Fig. S3:**
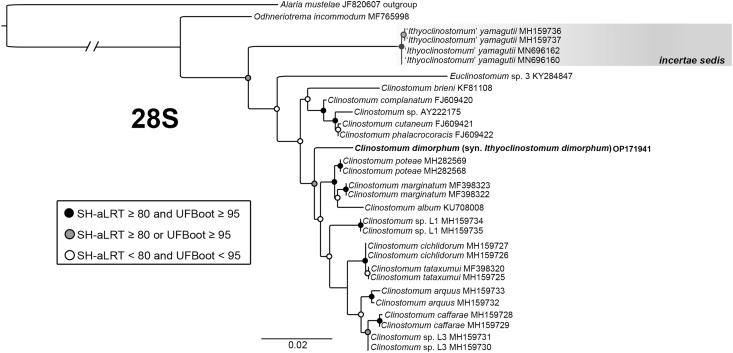
Maximum likelihood phylogram based on the 28S dataset of *Clinostomum dimorphum* (in bold) and selected Clinostomidae. Clade formed by isolates of ‘*Ithyoclinostomum*’ *yamagutii* (*incertae sedis*) is highlighted in grey. Taxon names are followed by GenBank accession numbers. Branch length scale bar indicates number of substitutions per site.

**Supplementary Fig. S4:**
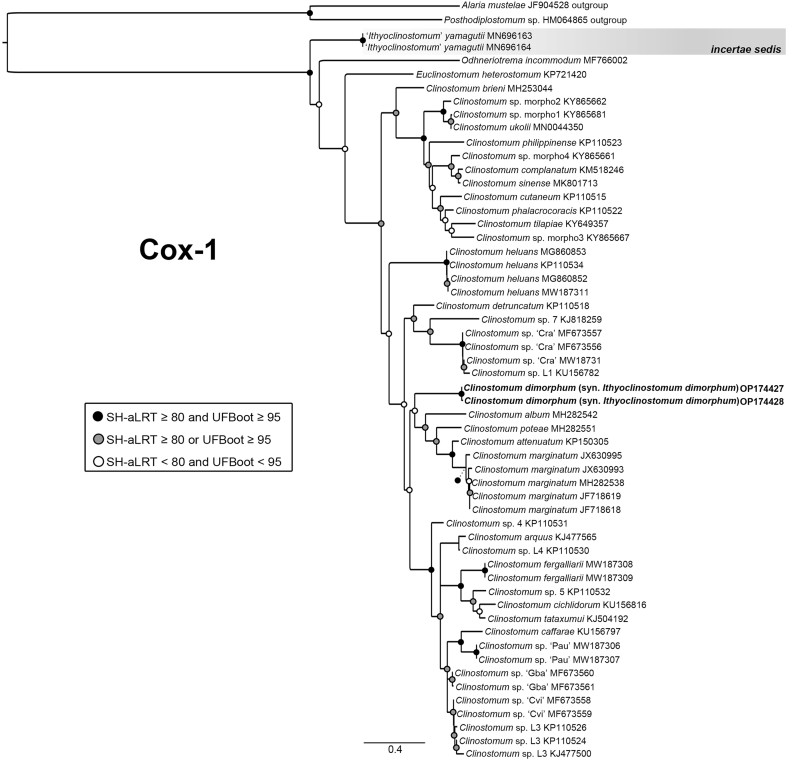
Maximum likelihood phylogram based on the cox1 dataset of *Clinostomum dimorphum* (in bold) and selected Clinostomidae. Clade formed by isolates of ‘*Ithyoclinostomum*’ *yamagutii* (*incertae sedis*) is highlighted in grey. Taxon names are followed by GenBank accession numbers. Branch length scale bar indicates number of substitutions per site.

The trees resulting from concatenated (Fig. 2) and the single-gene (Supplementary Figs. S2–S4) phylogenetic analyses show unequivocally that *Ithyoclinostomum dimorphum* is deeply nested among the species-rich genus *Clinostomum*, clustering with the New World species but with an uncertain position in this clade. The only other species of *Ithyoclinostomum* known, *I*. *yamagutii*, appear in all tree reconstructions as an earlier diverging, independent lineage, yet its position in relation to other clinostomids varies among datasets. Whereas in the partitioned *cox*1 alone *I*. *yamagutii* is the earliest diverging taxon, in all remaining phylogenies it is placed as sister (statistical support also varies) of a large clade including representatives of *Euclinostomum* and *Clinostosmum*; in the latter topology, *Odhneriotrema incommodum* (Leidy, 1856) appears as sister to all other clinostomid taxa. Regarding the classical, morphology-based subfamilial classification of Clinostomidae, the monophyly of Nephrocephalinae cannot be confidently assessed as sequences of *Nephrocephalus* spp. are missing, while the unique molecular signature of isolates of the monogeneric subfamily Euclinostominae, i.e, represented only by *Euclinostomum*, makes its monophyly plausible. Lastly, the reciprocal monophyly of Clinostominae and Ithyoclinostominae was not supported by any of the analyses with the nesting of *I*. *dimorphum* within *Clinostomum*. Additionally, *cox*1 data confirmed the conspecificity between metacercariae from different fish species (0.9% molecular divergence, i.e., six base pairs of divergence at the 3rd codon position but with identical amino acids according to the trematode translational code).

Phylogenetic results obtained in this study reveals the necessity of the following nomenclatural acts (i) synonymization of *Ithyoclinostomum* Witenberg, 1926 with *Clinostomum* Leidy, 1856 (ii) re-establishment of *Clinostomum dimorphum* (Diesing, 1850), (iii) amendment of the genus *Clinostomum*, (iv) synonymization of Ithyoclinostominae Yamaguti, 1958 with Clinostominae Lühe, 1901. Such nomenclatural acts are formally proposed below.

### 3.1 Taxonomic summary

Subfamily Clinostominae Lühe, 1901.

(= Ithyoclinostominae Yamaguti, 1958 – new synonym).

Genus *Clinostomum* Leidy, 1856.

Diagnosis: As in Kanev et al. (2002), updated (broader concept) by Caffara et al. (2019), but with the following change: Body middle-sized to very large (5–100 mm long), slender to stout, may be attenuated anteriorly. Space between ventral sucker and anteriormost vitelline follicles may be void of organs.

*Clinostomum dimorphum* (Diesing, 1850) Braun,1901.

(new synonym: *Ithyoclinostomum dimorphum*).

*Hosts*: *Hoplias malabaricus* (Bloch, 1794) and *Hoplias intermedius* (Günther, 1864).

*Prevalence of infection*: 1/1 (100%) for *H. malabaricus*, and 1/11 (9%) for *H. intermedius*.

*Intensity of infection*: 5 (*H. malabaricus*) and 1 (*H. intermedius*).

*Site of infection*: encysted at gill, heart and pericardial cavity (*H. malabaricus*), and liver (*H. intermedius*).

*Locality*: Doce River, municipalities of Colatina (19°31′57.9"S; 40°38′09.0"W) (*H. malabaricus*) and Baixo Guandu (19°30′17.8"S; 41°01′26.6"W (*H. intermedius*), State of Espirito Santo, Brazil.

*Representative DNA sequences*: Two nearly complete sequences of the ribosomal gene cluster ITS1-5.8S-ITS2 (length 970 bp ex *H*. *malabaricus* and 979 bp ex *H*. *intermedius*; identical in their overlapping range); a partial sequence of the 28S gene (length 1253 bp ex *H*. *malabaricus*); and two partial *cox*1 sequences (length 666 bp ex both *Hoplias* species; 0.9% of nucleotide divergence).

*Voucher material*: Four whole-mounted metacercariae (2 hologenophores and 2 paragenophores) deposited at the Collection of Trematodes of the Universidade Federal de Minas Gerais (UFMG-TRE 125–126).

Data presented in this study reflects the complexity and problematic nature of the morphologically based classification involving clinostomids, complementing the recent study of Caffara et al. (2019) that molecularly confirmed the placement of the large-sized *Clinostomoides brieni* Dollfus, 1950 within *Clinostomum*; besides the large size, *C*. *brieni* further differs from the 'typical' *Clinostomum* species by having the genital pore post-testicular and the whole genital complex extremely close to the posterior end. These findings depict an opposing scenario to recent molecular phylogenetic studies (Woodyard et al., 2017; Rosser et al., 2020) that have endorsed previous morphological arrangement for the family (Kanev et al., 2002). Moreover, our data raise doubts concerning which morphological features are informative at higher-level classification schemes. Size, for instance, has been considered a major taxonomic criterion in most helminth classifications and was the main differential trait for the proposition of *Ithyoclinostomum* and Ithyoclinostominae (Yamaguti, 1958; Kanev et al., 2002). However, despite the giant and unbelievable difference in body length between type species of the genus, *Clinostomum complanatum* (Rudolphi, 1814) and *C. dimorphum* (∼5 mm *vs* up to 100 mm; more than 2000% larger), the phylogenetic analyses herein presented revealed there is no support for these species be considered as belonging to distinct clinostomid genera. To the best of our knowledge, such an enormous difference among congeneric trematodes, has never been reported for any other genus of trematodes, making the quote "looks can deceiving" well appropriated to describe our findings.

In our phylogenetic analyses, the recently described *Ithyoclinostomum yamagutii* presented as an early diverging and isolated lineage from other clinostomids, as previously shown by Briosio-Aguilar et al. (2019) and Rosser et al. (2020). On one hand, these authors assigned their specimens to *Ithyoclinostomum* primarily based on: the large body size, the position of the cirrus-sac (pre-testicular), the testes shape (deeply lobed), the position of gonads in the posterior fourth of the body, and the large free area (void of any internal organ) between the ventral sucker and anterior testis; these features are found in the type species of the genus, *Ithyoclinostomum dimorphum,* now *C. dimorphum*. On the other hand, they assertively comment that obtaining molecular data from the type species of *Ithyoclinostomum* is crucial either to confirm or reject their hypothesis as well as to assess the interrelationships among other clinostomid genera, which was done in this study. The phylogenies and the high molecular divergences verified between *C. dimorphum* and *I*. *yamagutii* (7.6% in 28S, 12.6% in ITS, and 18.6% in *cox*1) reveal unequivocally these species are not congeneric. Based on the synonym between *Clinostomum* and *Ithyoclinostomum* required after our results, the taxon studied by Briosio-Aguilar et al. (2019) and Rosser et al. (2020) must be transferred to another genus, but for the sake of nomenclatural stability in the group, we provisionally retained the species in the non-natural *Ithyoclinostomum* [using quotation marks ('*Ithyoclinostomum*' or '*I*.') to refer to the genus] until key taxa (see below) are sequenced and compared with available data (see Table S1).

It is possible that '*I*.' *yamagutii* may deserve a new clinostomid genus to accommodate it. However, given the complex scenario raised in the morphology-based taxonomy of Clinostomidae (traditionally used features such as body size and arrangement of the genital system were found unreliable for higher-level classification purposes), we chose not to erect a new genus for '*I*.' *yamagutii* until molecular data on other clinostomid genera are available, especially for *Clinostomatopsis sorbens* (Braun, 1899), the type and only species of the genus *Clinostomatopsis* Dollfus, 1932 (Kanev et al., 2002). This clinostomid genus is the only one known from birds without molecular data available. Our conservative approach is also justified due to the morphological similarities among *'I.' yamagutii* and *C*. *sorbens* that include: gonads located toward the posterior extremity of the body and the presence of deeply lobed or irregular-shaped testes. Given the likely plesiomorphic/homoplastic nature of morphological traits otherwise used to differentiate subfamilies and genera (e.g., genital pore position and anterior extension of vitellaria — see Caffara et al., 2019; present results), we also opt by not to transfer '*I*.' *yamagutii* to the genus *Clinostomatopsis*.

Despite the contribution of molecular phylogeny for proposing a more natural classification that reflects the evolution of the members of the family Clinostomidae, some key taxa (type genera and species) still need to be sequenced to test the traditional morphology-based classification system. For instance, in reptile clinostomids, *O. incommodum* was sequenced based on worms found in alligators from USA (Woodyard et al., 2017). However, no molecular data is available for *Odhneriotrema microcephala* (Travassos, 1922), the type species of the genus *Odhneriotrema* Travassos, 1928, described from Brazil. Thus, despite morphological similarities between these species, the possibility they correspond to distinct genera cannot be ruled out. Such delay in the generation of molecular data is also verified for the genus *Nephrocephalus* Odhner, 1902, found in African reptiles, which is the type genus of Nephrocephalinae Travassos, 1928. Therefore, the phylogenetic position of its members in relation to the other clinostomids is unknown. A similar scenario can be found even at the species level, which can be evidenced by the case of *Clinostomum marginatum* (Rudolphi, 1819). Despite the fact that this species has been sequenced from isolates obtained from birds and fish in North America (Caffara et al., 2011; Rosser et al., 2017), its specific assignment should be confirmed once isolates from the type locality in Brazil, are sequenced (Pinto et al., 2015; Montes et al., 2021).

Overall, the taxonomic issues raised in this study highlight the importance of the availability of molecular data for the type species of trematode genera described from South America. Since the XIX century, dozens of trematode genera were proposed from this continent, initially from material collected in Brazil by the naturalist Johann Natterer and described by European helminthologists. Later in the XX century, renowned trematode taxonomists such as Szidat, Travassos, Thatcher, and their disciples described several other trematode genera, especially in Brazil and Argentina (Cribb and Bray, 2011). Unfortunately, most of the type species of these genera have not been sequenced so far. The lack of sequences for these species may be a reflection of limited access to sequencing methodologies compared with developed countries. This bias is verified in different groups of trematodes (Poulin and Jorge 2019), and despite advances verified in the last few years, most type-species of trematodes described from South America have not yet been sequenced. Such information is essential for more robust and natural classification and specially to support an inter- or transcontinental distribution of species assigned to the same genus.

## Declaration of competing interest

The authors declare no conflicts of interest.
