# Supplementary material for: Size does not matter: molecular phylogeny reveals one of the largest trematodes from vertebrates, the enigmatic Ithyoclinostomum dimorphum, as a species of Clinostomum (Trematoda: Clinostomidae)
Source: Int J Parasitol Parasites Wildl. 2022 Aug 24;19:84–8. doi: 10.1016/j.ijppaw.2022.08.002 (PMC9449640; doi:10.1016/j.ijppaw.2022.08.002)
Supplement: Multimedia component 1 [file mmc1.docx]

**Table S1.** List of valid clinostomid species sorted by their subfamilial classification. Shaded cells correspond to the availability of formally published data for most densely sampled molecular markers retrieved from the GenBank database as of June 2022.

| **Taxon** | **Alternative GenBank entry name(s)** | **Molecular markers** | | |
| --- | --- | --- | --- | --- |
|  |  | **ITS** | **28S** | ***cox*1** |
| **CLINOSTOMINAE** |  |  |  |  |
| ***Clinostomatopsis*** Dollfus, 1932 |  |  |  |  |
| *Clinostomatopsis sorbens*** (Braun, 1899) | **—** | **-** | **-** | *-* |
| ***Clinostomum**** Leidy, 1856 |  |  |  |  |
| *Clinostomum album* Rosser *et al*.*,* 2017 | **—** | **+** | **+** | **+** |
| *Clinostomum arquus* Sereno-Uribe *et al*., 2018 | *Clinostomum* sp. 3 or lineage 4 | **+** | **+** | **+** |
| *Clinostomum attenuatum* Cort, 1913 | **—** | **+** | **-** | **+** |
| *Clinostomum australiense* Johnston, 1916 | **—** | **-** | **-** | *-* |
| *Clinostomum brieni* (Dollfus, 1950) | *Clinostomoides brieni* | **+** | **-** | **+** |
| *Clinostomum caffarae* Sereno-Uribe *et al*.*,* 2018 | *Clinostomum* sp. lineage 2 | **+** | **+** | **+** |
| *Clinostomum cichlidorum* Sereno-Uribe *et al*.*,* 2018 | *Clinostomum* sp. lineage 5 | **+** | **+** | **+** |
| *Clinostomum complanatum*** (Rudolphi, 1814) | **—** | **+** | **+** | **+** |
| *Clinostomum cutaneum* Paperna, 1964 | **—** | **+** | **+** | **+** |
| *Clinostomum detruncatum* Braun, 1899 | **—** | **+** | **-** | **+** |
| *Clinostomum fergalliarii* Montes *et al.,* 2021 |  | **-** | **-** | **+** |
| *Clinostomum heluans* Braun, 1899 | *Clinostomum* sp. 6 | **+** | **-** | **+** |
| *Clinostomum hornum* Nicoll, 1914 | **—** | **-** | **-** | *-* |
| *Clinostomum intermedialis**** Lamont, 1920 | **—** | **-** | **-** | *-* |
| *Clinostomum kassimovi* Vaidova et Feizullaev, 1958 | **—** | **-** | **-** | *-* |
| *Clinostomum marginatum* (Rudolphi, 1819) sensu lato | **—** | **+** | **+** | **+** |
| *Clinostomum phalacrocoracis* Dubois, 1930 | **—** | **+** | **+** | **+** |
| *Clinostomum philippinense* Velasquez, 1959 | **—** | **+** | **-** | **+** |
| *Clinostomum poteae* Rosser *et al*.*,* 2018 | **—** | **+** | **+** | **+** |
| *Clinostomum sinense* Locke *et al*.*,* 2019 | *Clinostomum* sp. 8 | **+** | **-** | **+** |
| *Clinostomum taxaxumui* Sereno-Uribe *et al*., 2013 |  | **+** | **+** | **+** |
| *Clinostomum tilapiae* Ukoli, 1966 | **—** | **+** | **-** | **+** |
| *Clinostomum ukolii* Caffara *et al.,* 2020 | *Clinostomum* morphotype 1 | **+** | **-** | **+** |
| *Clinostomum wilsoni* Matthews et Cribb, 1998 | **—** | **-** | **-** | *-* |
| **EUCLINOSTOMINAE** |  |  |  |  |
| ***Euclinostomum**** Travassos, 1928 |  |  |  |  |
| *Euclinostomum ardeolae* El-Naffar et Khalifa, 1981 | **—** | **-** | **-** | *-* |
| *Euclinostomum clarias* (Dubois, 1930) | **—** | **-** | **-** | *-* |
| *Euclinostomum dollfusi* Fischthal et Kuntz, 1963 | **—** | **-** | **-** | *-* |
| *Euclinostomum heterostomum*** (Rudolphi, 1809) | **—** | **+ +** | **+** | **+** |
| *Euclinostomum multicaecum* Tubangui et Masilungan, 1935 | **—** | **-** | **-** | *-* |
| **ITHYOCLINOSTOMINAE** |  |  |  |  |
| ***Ithyoclinostomum**** Witenberg, 1926 | **—** |  |  |  |
| *Ithyoclinostomum dimorphum*** (Diesing, 1850) | **—** | **-** | **-** | *-* |
| *Ithyoclinostomum yamagutii* Rosser *et al.,* 2020 | *Ithyoclinostomum* sp. | **+** | **+** | **+** |
| **NEPHROCEPHALINAE** |  |  |  |  |
| ***Nephrocephalus**** Odhner, 1902 |  |  |  |  |
| *Nephrocephalus bagriincapsulatus* (Wedl, 1861) | **—** | **-** | **-** | *-* |
| *Nephrocephalus gerberi* (Baer, 1959) | **—** | **-** | **-** | *-* |
| *Nephrocephalus sessilis*** Odhner, 1902 | **—** | **-** | **-** | *-* |
| ***Odhneriotrema*** Travassos, 1928 |  |  |  |  |
| *Odhneriotrema incommodum* (Leidy, 1856) | **—** | **+** | **+** | **+** |
| *Odhneriotrema microcephala*** (Travassos, 1922) | **—** | **-** | **-** | *-* |

*type-genus of recognized subfamilies; ** type-species; ***this species was assigned to the genus *Clinostomatopsis* (as *C*. *intermedialis*) by Lunaschi and Drago (2009) but they neither proposed the new combination formally nor presented any justification for this taxonomic action.

**References**

Lunaschi, L.I., Drago, F. B., 2009. Digenean parasites of six species of birds from Formosa Province, Argentina. Rev. Mex. Biodivers. 80, 39–46.
