# Supplementary material for: Size does not matter: molecular phylogeny reveals one of the largest trematodes from vertebrates, the enigmatic Ithyoclinostomum dimorphum, as a species of Clinostomum (Trematoda: Clinostomidae)
Source: Int J Parasitol Parasites Wildl. 2022 Aug 24;19:84–8. doi: 10.1016/j.ijppaw.2022.08.002 (PMC9449640; doi:10.1016/j.ijppaw.2022.08.002)
Supplement: Multimedia component 2 [file mmc2.docx]

**Table S2**. Measurements of molecular voucher specimens of *Clinostomum dimorphum* (= *Ithyoclinostomum dimorphum*) found in *Hoplias malabaricus* and *Hoplias intermedius* at the Doce River, Espírito Santo State, Brazil. Data are given in mm.

| Host |  | *Hoplias* | *Hoplias* | *Hoplias* |
| --- | --- | --- | --- | --- |
|  |  | *malabaricus* | *intermedius* | *intermedius* |
| Locality |  | Baixo Guandu | Colatina | Colatina |
| Molecular voucher |  | Hologenophore | Hologenophore | Paragenophore |
|  |  |  |  |  |
| Body | L | 45 | 32 | 38 |
|  | W | 4.15 | 3.83 | 3.74 |
| Oral sucker | L | 0.36 | - | 0.46 |
|  | W | 0.59 | - | 0.5 |
| Ventral sucker | L | 1.71 | 1.46 | 1.66 |
|  | W | 1.46 | 1.37 | 1.46 |
| Anterior testis | L | 0.19 | 0.15 | 0.27 |
|  | W | 1.00 | 0.97 | 1.13 |
| Posterior testis | L | 0.22 | 0.18 | 0.27 |
|  | W | 1.12 | 0.91 | 1.113 |
| Ovary | L | - | 0.21 | 0.25 |
|  | W | - | 0.14 | 0.11 |
|  |  |  |  |  |
